# Supplementary figures and images for: TRIM59: A potential diagnostic and prognostic biomarker in human tumors
Source: PLoS One. 2021 Sep 17;16(9):e0257445. doi: 10.1371/journal.pone.0257445 (PMC8448305; doi:10.1371/journal.pone.0257445)

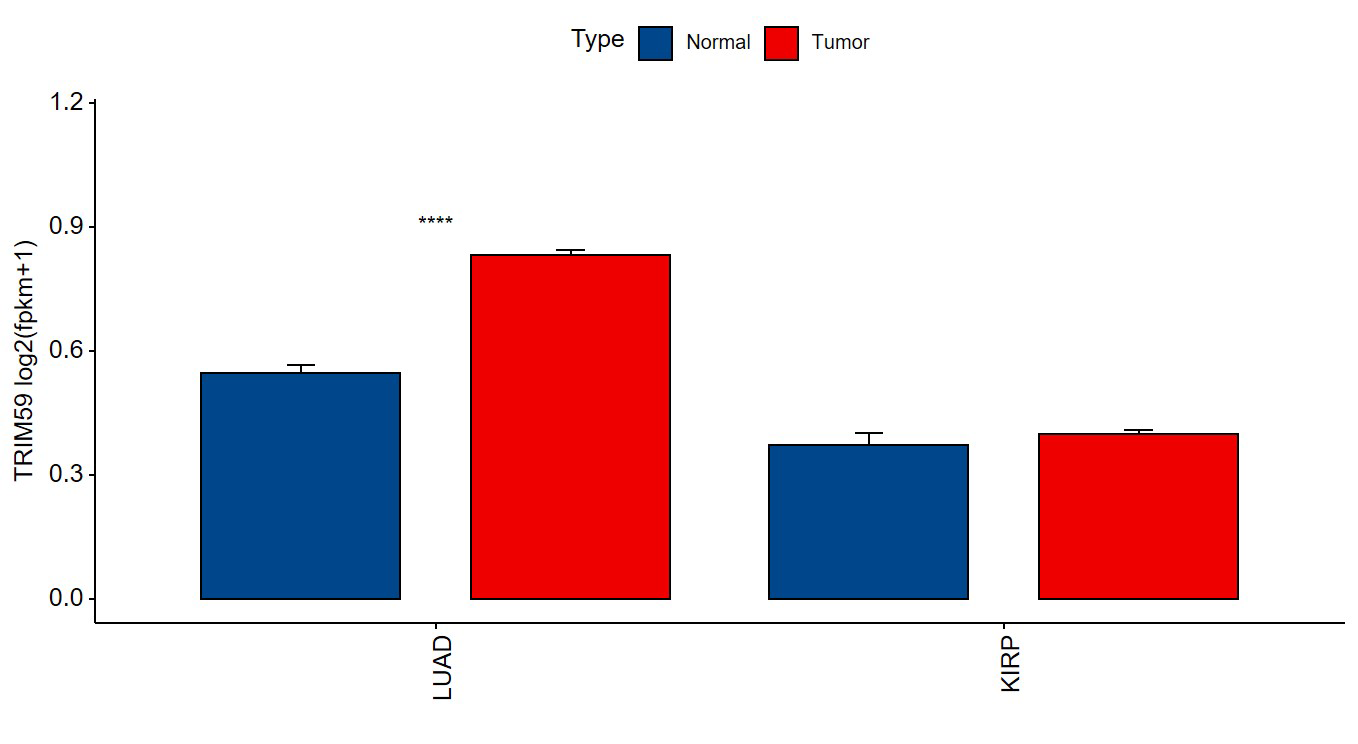

Supplement: S1 Fig — (TIF) [file pone.0257445.s001.tif]
